# Human renal fibroblasts are strong immunomobilizers during a urinary tract infection mediated by uropathogenic *Escherichia coli*

**DOI:** 10.1038/s41598-019-38691-8

**Published:** 2019-02-19

**Authors:** Kristin Klarström Engström, Boxi Zhang, Isak Demirel

**Affiliations:** 1 Department of Clinical trial unit, Faculty of Medicine and Health, Örebro University, Örebro, Sweden; 2 Department of Physiology and Pharmacology, Karolinska Institutet, Stockholm, Sweden; 3 iRiSC - Inflammatory Response and Infection Susceptibility Centre, Faculty of Medicine and Health, Örebro University, Örebro, Sweden; 4 School of Medical Sciences, Örebro University, Örebro, Sweden

## Abstract

To prevent the onset of urosepsis and reduce mortality, a better understanding of how uropathogenic *Escherichia coli* (UPEC) manages to infiltrate the bloodstream through the kidneys is needed. The present study elucidates if human renal interstitial fibroblasts are part of the immune response limiting a UPEC infection, or if UPEC has the ability to modulate the fibroblasts for their own gain. Microarray results showed that upregulated genes were associated with an activated immune response. We also found that chemokines released from renal fibroblasts upon a UPEC infection could be mediated by LPS and triacylated lipoproteins activating the TLR2/1, TLR4, MAPK, NF-κB and PKC signaling pathways. Furthermore, UPEC was also shown to be able to adhere and invade renal fibroblasts, mediated by the P-fimbriae. Furthermore, it was found that renal fibroblasts were more immunoreactive than renal epithelial cells upon a UPEC infection. However, both renal fibroblasts and epithelial cells were equally efficient at inducing neutrophil migration. In conclusion, we have found that human renal fibroblasts can sense UPEC and mobilize a host response with neutrophil migration. This suggests that renal fibroblasts are not only structural cells that produce and regulate the extracellular matrix, but also highly immunoreactive cells.

## Introduction

Urinary tract infection (UTI) is one of the most common infections that affects human beings. Different types of bacteria can cause UTI, but the majority of the cases are caused by uropathogenic *Escherichia coli* (UPEC)^1^. The majority of the UTI are local infections, but in some cases complicated UTI develops, which can result in pyelonephritis, bacteremia and urosepsis. Urosepsis accounts for a quarter of all sepsis cases and can be a life-threatening condition that must be treated immediately^2,3^. Worldwide, more than 30 million people suffer from sepsis annually with a mortality rate of 30–40%^4,5^. A prompt diagnosis and adequate treatment is critical during sepsis, as the risk of dying increases for each passing hour without adequate treatment. To prevent the onset of urosepsis and reduce mortality, a better understanding of how bacteria like UPEC manages to infiltrate the bloodstream through the kidneys is needed, likewise how UPEC modulates the immune cells in the kidneys and bloodstream to its advantage. Fibroblasts have traditionally been seen as structural cells that produce and regulate the extracellular matrix in tissues. However, recent discoveries have shown that fibroblasts are important immunoreactive cells. They can recognize pathogens and produce cytokines and chemokines which recruit leukocytes to the infected tissue. In addition, it has also been shown that fibroblasts interact with infiltrated and tissue-resident immune cells, such as monocytes, neutrophils, dendritic cells and T cells by modulating their immune response^6,7^. However, fibroblasts from different anatomical sites have been found to have various expression phenotypes, making it hard to generalize findings between different tissue-specific fibroblasts^8–10^. To the best of our knowledge, no studies have investigated the host-pathogen interaction between primary human renal fibroblasts and UPEC. After breaching the renal epithelium, but before reaching the bloodstream, UPEC will be in direct contact with interstitial renal fibroblasts. The outcome of this interaction is largely unknown. Will the renal fibroblasts contribute to the host response and limit the spread of the infection? Or will UPEC modulate the fibroblast responses to persist and spread to the bloodstream? Hence the need of understanding the interaction between UPEC and renal fibroblasts. We and others have shown that UPEC has the ability to modulate the immune response in the urinary tract via various virulence factors such as type-1 fimbriae, P-fimbriae, α-hemolysin, IrmA and TcpC to colonize the urinary tract^11–14^. However, which virulence factors UPEC utilizes in the interaction with renal fibroblasts is unknown. Our aim was to elucidate if human renal fibroblasts are a part of the immune response limiting the UPEC infection, or if UPEC has the ability to modulate the fibroblasts for its persistence and spreading.

## Results

### Gene expression alterations in UPEC infected renal fibroblasts

A microarray analysis was performed on total RNA isolated from primary human renal fibroblasts infected with the UPEC strain CFT073. In total 1196 gene entities were upregulated and 509 gene entities (Supplementary Table S1) were downregulated (corrected p < 0.05) with at least a ≥2 fold change compared to unstimulated renal fibroblasts after 6 hours. The thirty highest upregulated and downregulated gene entities are presented in Tables 1 and 2, respectively. In order to validate the microarray results, real time qPCR was conducted on four significantly upregulated (IL-1β, NOD2, CXCL10 and CXCL9) and four significantly downregulated (PTCH1, TET1, PLCB2 and CPEB1) genes compared to unstimulated renal fibroblasts. In agreement with the microarray analysis, a significant upregulation of IL-1β, CXCL10 and CXCL9 was observed. We also found that PTCH1, TET1, PLCB2 and CPEB1 were significantly downregulated compared to unstimulated renal fibroblasts (Table 3).

**Table 1:** CFT073 induced gene upregulation compared to unstimulated renal fibroblasts.

Gene symble	Fold change	Description
CXCL10	16858	Homo sapiens chemokine (C-X-C motif) ligand 10
CXCL9	6485	Homo sapiens chemokine (C-X-C motif) ligand 9
CXCL11	3525	Homo sapiens chemokine (C-X-C motif) ligand 11
OASL	2200	Homo sapiens 2′-5′-oligoadenylate synthetase-like
RSAD2	1645	Homo sapiens radical S-adenosyl methionine domain containing 2
CCL5	1610	Homo sapiens chemokine (C-C motif) ligand 5
XLOC_l2_000297	1507	BROAD Institute lincRNA (XLOC_l2_000297), lincRNA
CD38	611	Homo sapiens CD38 molecule
GBP4	592	Homo sapiens guanylate binding protein 4
CSF3	587	Homo sapiens colony stimulating factor 3
CCL8	532	Homo sapiens chemokine (C-C motif) ligand
GBP1P1	498	Homo sapiens guanylate binding protein 1, interferon-inducible pseudogene 1
IFIT2	440	Homo sapiens interferon-induced protein with tetratricopeptide repeats 2
CMPK2	439	Homo sapiens cytidine monophosphate (UMP-CMP) kinase 2
SSTR2	384	Homo sapiens somatostatin receptor 2
HERC5	380	Homo sapiens HECT and RLD domain containing E3 ubiquitin protein ligase 5
KIF5C	361	Homo sapiens kinesin family member 5C
TNFSF13B	273	Homo sapiens tumor necrosis factor (ligand) superfamily, member 13b
HRASLS2	268	Homo sapiens HRAS-like suppressor 2
IDO1	255	Homo sapiens indoleamine 2,3-dioxygenase 1
NOD2	243	Homo sapiens nucleotide-binding oligomerization domain containing 2
C16orf47	242	Homo sapiens chromosome 16 open reading frame 47
GPR84	222	Homo sapiens G protein-coupled receptor 84
GBP5	218	Homo sapiens guanylate binding protein 5
CIITA	211	Homo sapiens class II, major histocompatibility complex, transactivator
OAS1	179	Homo sapiens 2′-5′-oligoadenylate synthetase 1
UBD	175	Homo sapiens ubiquitin D
GCH1	166	Homo sapiens GTP cyclohydrolase 1
CCL20	166	Homo sapiens chemokine (C-C motif) ligand 20
TBC1D1	141	Homo sapiens TBC1 (tre-2/USP6, BUB2, cdc16) domain family, member 1

**Table 2:** CFT073 induced gene downregulation compared to unstimulated renal fibroblasts.

Gene symble	Fold change	Description
KIT	−24,3	Homo sapiens v-kit Hardy-Zuckerman 4 feline sarcoma viral oncogene homolog
PTCH1	−13,1	Homo sapiens patched 1
TET1	−9,3	Homo sapiens tet methylcytosine dioxygenase 1
CEBPA-AS1	−8,6	Homo sapiens CEBPA antisense RNA 1
PLCB2	−8,2	Homo sapiens phospholipase C, beta 2
EEPD1	−7,7	Homo sapiens endonuclease/exonuclease/phosphatase family domain containing 1
CPEB1	−7,6	Homo sapiens cytoplasmic polyadenylation element binding protein 1
FIGNL2	−7,0	Homo sapiens fidgetin-like 2
GRASP	−6,9	Homo sapiens GRP1 (general receptor for phosphoinositides 1)-associated scaffold protein
TOX	−6,7	Homo sapiens thymocyte selection-associated high mobility group box
MKNK2	−6,6	Homo sapiens MAP kinase interacting serine/threonine kinase 2
STAM-AS1	−6,6	Homo sapiens STAM antisense RNA 1
SNHG22	−6,6	Homo sapiens small nucleolar RNA host gene 22
ADRA2A	−6,3	Homo sapiens adrenoceptor alpha 2A
RCOR2	−6,2	Homo sapiens REST corepressor 2
CEBPA	−6,1	Homo sapiens CCAAT/enhancer binding protein (C/EBP), alpha
SSTR1	−5,9	Homo sapiens somatostatin receptor 1
CSRNP3	−5,8	Homo sapiens cysteine-serine-rich nuclear protein 3
RGCC	−5,8	Homo sapiens regulator of cell cycle
MEX3B	−5,7	Homo sapiens mex-3 RNA binding family member B
THBD	−5,7	Homo sapiens thrombomodulin
ZNF518A	−5,6	zinc finger protein 518A
EPHB3	−5,6	Homo sapiens EPH receptor B3
GAS6-AS1	−5,6	Homo sapiens cDNA FLJ35543 fis, clone SPLEN2002957
E2F7	−5,5	Homo sapiens E2F transcription factor 7
KCNE3	−5,5	Homo sapiens potassium channel, voltage gated subfamily E regulatory beta subunit 3
CAMKK1	−5,5	Homo sapiens calcium/calmodulin-dependent protein kinase kinase 1, alpha
GAS1	−5,3	Homo sapiens growth arrest-specific 1
TRIM45	−5,3	Homo sapiens tripartite motif containing 45
SLC52A1	−5,3	Homo sapiens solute carrier family 52

**Table 3:** Quantitative real-time PCR data for CFT073 compared to unstimulated renal fibroblasts.

Gene symble	Fold change	Description
IL-1β	75 ± 23^a^	Interleukin 1, beta
NOD2	350 ± 126	Nucleotide-binding oligomerization domain containing 2
CXCL10	17910 ± 1327^a^	Chemokine (C-X-C motif) ligand 10
CXCL9	18249 ± 5777^a^	Chemokine (C-X-C motif) ligand 10
PTCH1	−7.9 ± 1.1^a^	Patched 1
TET1	−3.0 ± 0.4^a^	Tet methylcytosine dioxygenase 1
PLCB2	−1.6 ± 1.1^a^	Phospholipase C, beta 2
CPEB1	−9.5 ± 0.3^a^	Cytoplasmic polyadenylation element binding protein 1

n = 3.

^a^Significantly altered genes compared to unstimulated renal fibroblasts.

### Gene ontology and KEGG pathway analysis

**Figure 1:**
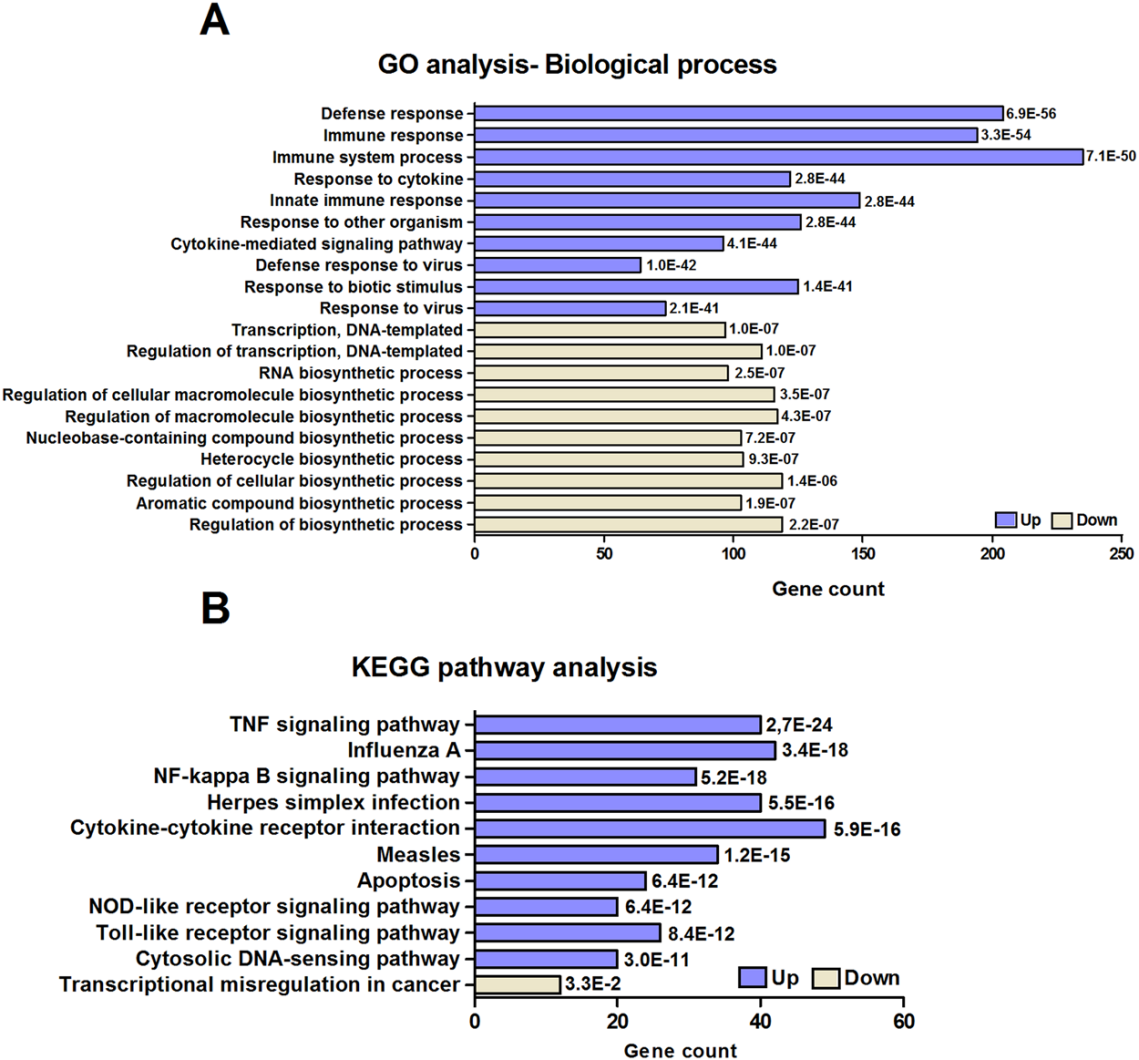
Gene ontology and KEGG pathway analysis for genes differentially expressed by renal fibroblasts infected with CFT073. The top ten enriched GO-biological processes (**A)** and KEGG pathways (**B**) in human renal fibroblasts after a CFT073 infection for 6 hours. Corrected p-values < 0.05. Each enriched Gene ontology is presented with respective false discovery rate.

Gene ontology analysis was conducted on significantly altered gene entities compared to unstimulated renal fibroblasts. In total, 674 upregulated (Supplementary Table S2) and 42 downregulated (Supplementary Table S3) gene ontologies were enriched. The top ten upregulated gene ontologies were defense response, immune response, immune system process, response to cytokine, innate immune response, response to other organism, cytokine-mediated signaling pathway, defense response to virus, response to biotic stimulus and response to virus. The top ten downregulated gene ontologies were transcription DNA-templated, regulation of transcription DNA-templated, RNA biosynthetic process, regulation of cellular macromolecule biosynthetic process, regulation of macromolecule biosynthetic process, nucleobase-containing compound biosynthetic process, heterocycle biosynthetic process, regulation of cellular biosynthetic process, aromatic compound biosynthetic process and regulation of biosynthetic process (Fig. 1A). Furthermore, we also found that the upregulated gene entities were significantly (corrected p < 0.05) enriched in 53 KEGG pathways (Supplementary Table S4) and the downregulated gene entities in 1 KEGG pathway (Supplementary Table S5). The top ten upregulated KEGG pathways were TNF signaling pathway, influenza A, NF-kappa B signaling pathway, Herpes simplex infection, cytokine-cytokine receptor interaction, measles, apoptosis, NOD-like receptor signaling pathway, Toll-like receptor signaling pathway and cytosolic DNA-sensing pathway. The downregulated KEGG pathway was transcriptional misregulation in cancer (Fig. 1B).

### IL-8 release from renal fibroblasts

**Figure 2:**
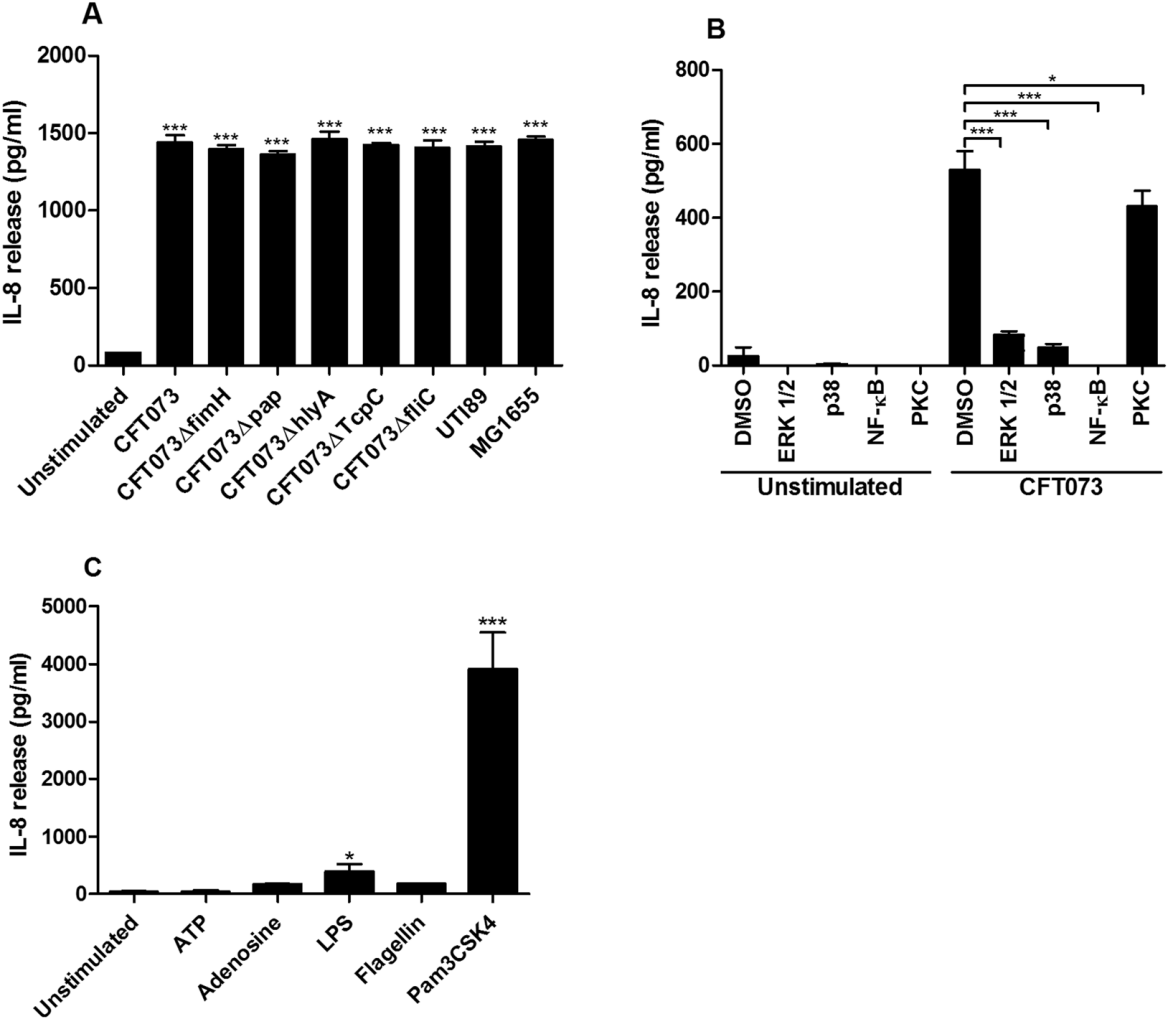
IL-8 release from renal fibroblasts. Primary human renal fibroblasts were stimulated with UTI89, MG1655, CFT073, CFT073Δpap, CFT073ΔfimH, CFT073ΔhlyA, CFT073ΔTcpC and CFT073ΔfliC at MOI 10 (**A**), adenosine triphosphate (100 µM, ATP), Adenosine (100 µM), lipopolysaccharide (1 µg/ml, LPS), flagellin (1 µg/ml) and Pam3CSK4 (1 µg/ml) for 6 hours. (**C**) The fibroblasts were also pre-incubated with DMSO (vehicle), p38 inhibitor SB203580 (10 µM), ERK1/2 inhibitor PD98059 (10 µM), NF-κB inhibitor BAY 11-7082 (5 µM) and the PKC inhibitor bisindolylmaleimide I (10 µM) for 1 hours prior to CFT073 stimulation for 6 hours at MOI 10. (**B**) IL-8 release was analyzed after stimulation. Data are presented as mean ± SEM (n = 4 independent experiments). Asterisks denote statistical significance compared to respective unstimulated control cells (*p < 0.05, ***p < 0.001).

As the microarray analysis showed that several chemokines were highly induced by CFT073, experiments were performed to examine the involvement of various UPEC virulence factors on IL-8 release using P-fimbriae (*pap*), type-1 fimbriae (*fimH*), α-hemolysin (*hlyA*), TIR homologous protein (*TcpC*), flagellin (*fliC*) CFT073 deletion mutants. These experiments showed that neither the P-fimbriae, type-1 fimbriae, α-hemolysin, TcpC nor flagellin were responsible for the CFT073 induced IL-8 release from renal fibroblasts after 6 hours. No significant IL-8 release was observed after 3 hours of bacterial stimulation (data not shown). In addition, no difference in IL-8 release was observed between CFT073, UTI89 or MG1655 (Fig. 2A). Using the same bacterial setup, we also found that neither the P-fimbriae, type-1 fimbriae, α-hemolysin, TcpC nor flagellin were responsible for the CFT073 induced CXCL10 release from renal fibroblasts after 6 hours. However, we did show that MG1655, but not UTI89 induced a significantly higher CXCL10 release compared to CFT073 (Supplementary Fig. S1). Microarray analysis showed that neither the P-fimbriae, type-1 fimbriae nor α-hemolysin were responsible for any of the significantly altered genes induced by CFT073 compared to unstimulated fibroblasts (data not shown). We continued with evaluating which signaling pathways were involved in the CFT073-induced IL-8 release from renal fibroblasts. We found that inhibition of p38, ERK1/2, NF-κB and PKC resulted in significantly lower IL-8 release from renal fibroblasts compared to cells infected with CFT073 in the presence of the vehicle DMSO after 6 hours (Fig. 2B). In addition, we also showed that LPS and Pam3CSK4, but not ATP, Adenosine or flagellin could induce an increased IL-8 release from renal fibroblasts compared to unstimulated cells after 6 hours (Fig. 2C). Taken together, these results suggest that UPEC-induce chemokine release from renal fibroblasts could be mediated by several different signaling pathways.

### CFT073 adheres to and invades renal fibroblasts

**Figure 3:**
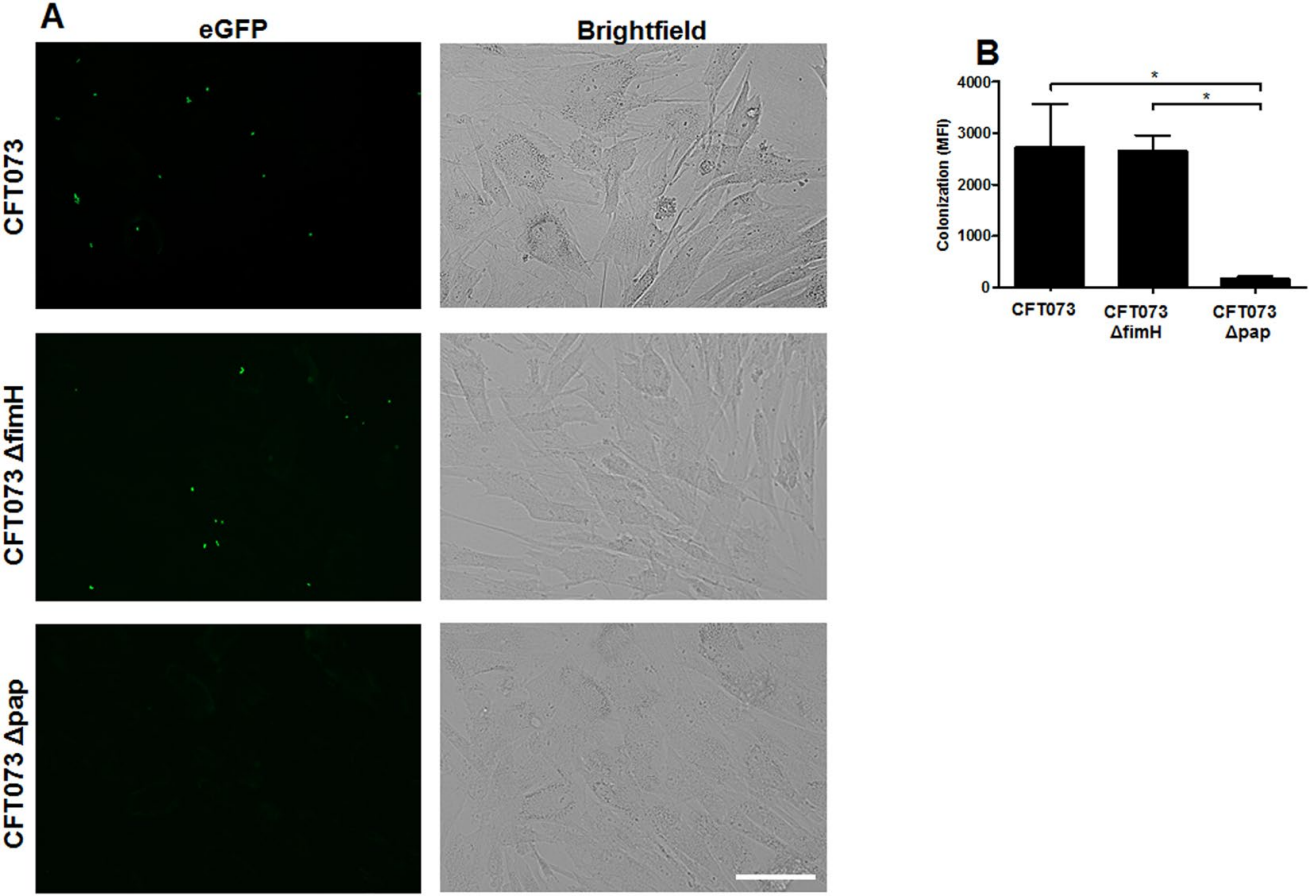
UPEC colonization of renal fibroblasts. Primary human renal fibroblasts were stimulated with CFT073, CFT073Δpap and CFT073ΔfimH expressing enhanced green fluorescence protein (eGFP) for 3 hours at MOI10 followed by evaluation of colonization (adhered and intracellular bacteria). (**A**,**B**) Colonization is quantified as mean fluorescence intensity (MFI). (**B**) Scale bar: 100 µm.

**Figure 4:**
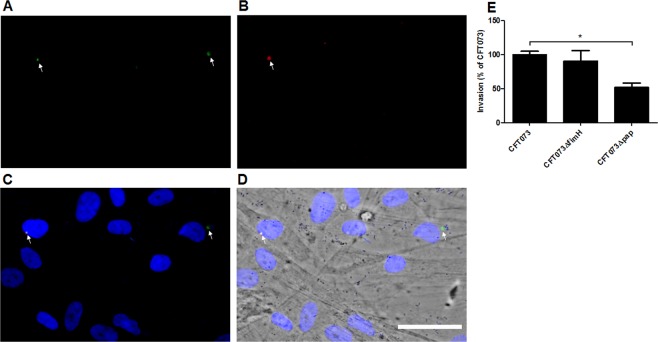
UPEC invasion of renal fibroblasts. Immunofluorescence staining of CFT073 following stimulation of renal fibroblasts for 3 hours. Staining of the fibroblasts cell nuclei was performed with DAPI and is shown in blue. (**C**,**D**) CFT073 are stained (**B**) in red (extracellular) prior to permeabilization, and (**A**) in green (extracellular and intracellular) after permeabilization. Merged image of (**A**,**B**) where intracellular bacteria are seen as green stain (arrows) and extracellular bacteria are shown as merged red and green (yellow) stain (arrows). (**C**,**D**) Stained CFT073 and cell nuclei is merged with bright field image of renal fibroblasts. (**D**) Representative images from three independent experiments are shown. Scale bar: 50 µm. Invasion was evaluated following stimulated with CFT073, CFT073Δpap and CFT073ΔfimH for 3 hours. (**E**) Invasion was presented as percentage of CFT073 invasion. Data are presented as mean ± SEM (n = 3 independent experiments). Asterisks denote statistical significance compared to CFT073 (*p < 0.05).

Further, evaluation of whether CFT073 could adhere and invade renal fibroblasts was performed. We found that CFT073 had the ability to adhere to renal fibroblasts (Fig. 3A,B) and that this adhesion was mediated by the P-fimbriae and not type-1 fimbriae (Fig. 3A,B). In addition, we also showed that the UPEC strain CFT073 was able to invade renal fibroblasts and that this invasion was partially mediated by the P-fimbriae and not type-1 fimbriae (Fig. 4A–E). We found that the maximum adhesion and invasion occurred after 3 hours. Together, our findings show that UPEC bacteria can adhere and invade renal fibroblast and this may protect the bacteria from the host immune response.

### Renal fibroblasts are more immunoreactive to CFT073 then renal epithelial cells

**Figure 5:**
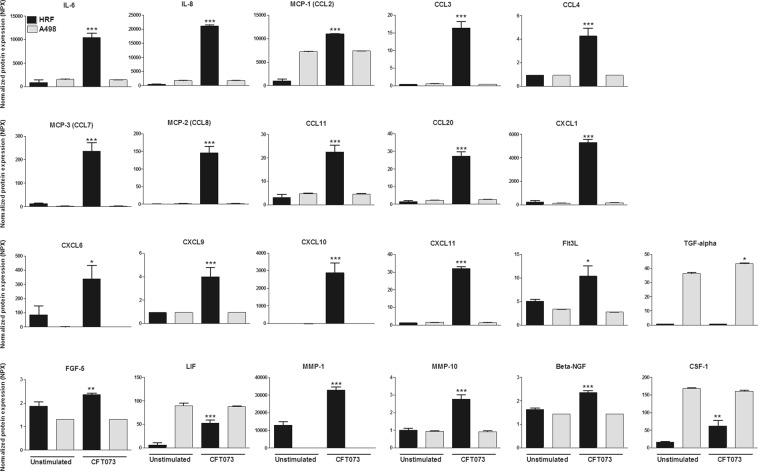
The release of inflammatory proteins from renal fibroblasts and renal epithelial cells upon a UPEC infection. Primary human renal fibroblasts (HRF) and human renal epithelial cells (A498) were stimulated with CFT073 at MOI 10 for 6 hours followed by the analysis of 92 inflammatory proteins simultaneously using the proximity extension assay on the Proseek Multiplex inflammation panel. The protein data is presented as normalized protein expression units (NPX) where a high value corresponds to high protein concentration. Data are presented as mean ± SEM (n = 3 independent experiments). Asterisks denote statistical significance compared to respective unstimulated control cells (*p < 0.05, *p < 0.01, ***p < 0.001).

We continued with comparing the immunoreactivity of renal fibroblasts and renal epithelial cells to a CFT073 infection with an Olink inflammation multiplex immunoassay (Supplementary Table S6). We found that 21 of 92 inflammatory-related proteins were significantly upregulated in renal fibroblasts after a CFT073 infection compared to unstimulated renal fibroblasts (Fig. 5). However, only one protein, TGF-alpha, was found to be significantly upregulated in renal epithelial cells after a CFT073 infection compared to unstimulated renal epithelial cells (Fig. 5). These findings show that renal fibroblasts are more immunoreactive than renal epithelial cells against a UPEC infection.

### Neutrophil migration is mediated by both renal fibroblasts and renal epithelial cells

**Figure 6:**
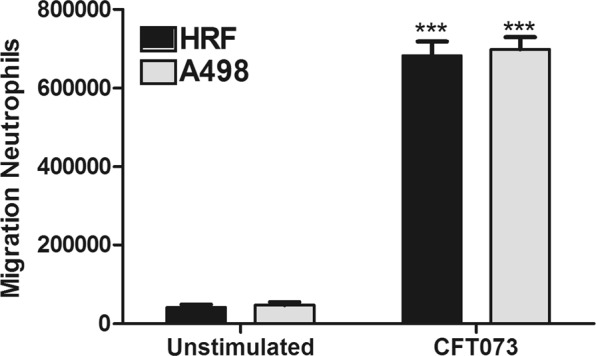
Neutrophil migration mediated by renal fibroblasts and renal epithelial cells. Primary human renal fibroblasts (HRF) and human renal epithelial cells (A498) were stimulated with CFT073 at MOI10 for 3 hours followed by neutrophil migration over a transwell membrane. Data are presented as mean ± SEM (n = 3 independent experiments). Asterisks denote statistical significance compared to respective unstimulated control cells (***p < 0.001).

We proceeded with investigating the ability of renal fibroblasts and renal epithelial cells to induce neutrophil migration in response to CFT073. We found that both renal epithelial cells and renal fibroblasts induced a significantly increased neutrophil migration compared to unstimulated cells after 3 hours (Fig. 6). However, we did not find any differences in neutrophil migration between the renal epithelial cells and renal fibroblasts (Fig. 6). These findings suggest that renal fibroblasts have the ability to recruit neutrophils in response to a UPEC infection.

## Discussion

To gain a better understanding on how UPEC interacts and manipulates the immune response in the kidneys, is important for preventing the bacteria from reaching the bloodstream. The vast majority of the research has so far been focused on studying the interaction between UPEC, renal epithelial cells and infiltrating leukocytes^15–18^. However, what role interstitial renal fibroblasts play in the progression of the infection has not been studied to our knowledge. Our aim for this study was to elucidate if human renal fibroblasts are a part of the immune response limiting the infection, or if UPEC has the ability to modulate the fibroblasts for its persistence and spreading.

We started by evaluating the global gene expression of primary human renal fibroblasts infected with the UPEC wild type strain CFT073. We found that CFT073 induced significant transcriptional alterations of a large number of genes in renal fibroblasts; 1196 upregulated and 509 downregulated gene entities. These findings were validated with real time RT-PCR for eight of the significantly altered genes (IL-1β, NOD2, CXCL10, PTCH1, TET1, PLCB2, CPEB1 and CXCL9). To the best of our knowledge, this is the first study that has investigated changes in the global gene expression profile of primary human renal fibroblasts infected with UPEC. Importantly, these data help us to understand the specific expression profile of renal fibroblasts evoked by UPEC and how this expression may differ from the profile of renal epithelial cells^19^ and infiltrated leukocytes^20,21^.

Gene ontology and KEGG pathway analysis showed that the upregulated genes were associated with an activated immune response. Key immunological pathways such as TNF signaling, NF-kappa B signaling, apoptosis, NOD-like receptor- and Toll-like receptor signaling pathway etc. were all enriched in the upregulated group. All mentioned pathways are known to be associated with pathogen recognition and the mobilization of the innate and adaptive immune response^22–24^. These findings are supported by the observed upregulation of several cytokines (e.g. IL-1, -6, -7, -15, -17C, -33) and chemokines (e.g. CXCL1, -2, -3, -4, -6, -8, -9, -10, -11 and CCL1, -2, -3, -5, -7, -8, -11, -13, -19, 20) in response to UPEC. Furthermore, we also found that the enriched gene ontologies and pathways for the downregulated genes were associated with transcription. A previous study has shown that *E*. *coli* is able to control host gene expression through modulation of RNA polymerase II^19^. However, further evaluation is needed to clarify if our observations are pointing towards the same conclusion. Taken together, these results suggests that renal fibroblasts are not only structural cells that produce and regulate the extracellular matrix, but also highly immunoreactive cells that can recognize UPEC and initiate an immune response.

It is well known that UPEC utilizes several different virulence factors like the P-fimbriae, type-1 fimbriae, α-hemolysin, TcpC and flagellin to colonize and modulate the immune response in the urinary tract. These virulence factors are known to be able to regulate the release of pro-inflammatory cytokines and chemokines, which we found to be highly induced by CFT073^25–27^. However, we found that neither the P-fimbriae, type-1 fimbriae, α-hemolysin, TcpC nor flagellin were responsible for the CFT073 induced IL-8 or CXCL10 release from renal fibroblasts. Additionally, this was validated by microarray, which showed that neither the P-fimbriae, type-1 fimbriae nor α-hemolysin were responsible for any of the significantly altered genes induced by CFT073 (data not shown). This suggest that neither the P-fimbriae, type-1 fimbriae, α-hemolysin, TcpC nor flagellin are important for the mobilized immune response induced by human renal fibroblasts. Furthermore, we also showed that the UPEC strain UTI89 and the non-pathogenic MG1655 strain could induce IL-8 and CXCL10 release from renal fibroblasts. Suggesting that this induction is not CFT073 or UPEC specific. To further explore the mechanisms behind the IL-8 release, we evaluated which signaling pathways CFT073 utilizes for the cytokine induction. We found that p38, ERK1/2, NF-κB and PKC were all involved in regulating the CFT073-induced IL-8 release from renal fibroblasts. The MAPK-pathway associated p38 and ERK1/2, NF-κB and PKC have all been shown to be important for the activation of the immune response in the kidneys^14,28,29^. Furthermore, we also showed that the TLR2/1 agonist Pam3CSK4 and the TLR4 agonist LPS induced an increased IL-8 release from renal fibroblasts, whereas the TLR5 agonist flagellin, the purinoceptor 1 agonist adenosine or the purinoceptor 2 agonist ATP did not. TLR2/1, TLR4, TLR5, purinoceptor 1 and purinoceptor 2^30–32^ have all been shown to be important in the host response against UPEC. Our results suggest that UPEC LPS and triacylated lipoproteins (TLR2/1 agonists) may be the pathogen associated molecular patterns (PAMPs) activating the renal fibroblasts. Taken together, these results suggest that UPEC-induced chemokine release from renal fibroblasts could be mediated by LPS and triacylated lipoproteins activating the TLR2/1, TLR4, MAPK, NF-κB and PKC signaling pathways.

Adhesion to epithelial cells is crucial for bacterial colonization of the urinary tract. UPEC is known to use the type-1 fimbriae for colonizing the bladder and the P-fimbriae for colonizing the kidneys^25^. In agreement with previous findings regarding the P-fimbriae and renal epithelial cells^33,34^, we found that UPEC utilizes the P-fimbriae, and not the type-1 fimbriae, for the adhesion/invasion of renal fibroblasts. The ability of UPEC to adhere and invade renal fibroblasts can be an evasion strategy utilized by UPEC to protect itself from infiltrated neutrophils and antibiotic treatment. Previous studies have associated intracellular UPEC with host evasion, antibiotic failure and recurrent UTI^35–38^. After the resolution of the immune response, UPEC may emerge out from the intracellular niche and continue the infection. This ability may be a piece of a puzzle explaining how UPEC, after breaching the renal epithelial layer, can persist in the renal interstitium and eventually reach the bloodstream.

By comparing the immune response of renal fibroblasts and renal epithelial cells upon a UPEC infection, a better understanding regarding what role the respective cell type has in clearing the infection might be reached. We found that renal fibroblasts are more immunoreactive than renal epithelial cells upon a UPEC infection, by using the Olink inflammatory multiplex panel. Out of 92 analyzed inflammatory-related proteins, 21 were significantly upregulated in fibroblasts and 1 was significantly upregulated in epithelial cells during a CFT073 infection compared to unstimulated control cells. Focusing on leukocyte migration, increased fibroblast release of several chemokines involved in neutrophil chemotaxis (IL-8, CXCL1, CXCL6), T- and natural killer (NK)-cell chemotaxis (CXCL9, CXCL10, CXCL11, CCL8), monocyte/macrophage chemotaxis (CCL2, CCL3, CCL4, CCL7), eosinophil and basophil chemotaxis (CCL11) and T-cells, B-cells and dendritic cells (DC) chemotaxis (CCL20) were found^39–41^. Our findings suggests that renal fibroblasts may have the ability to attract several different leukocytes during a UPEC infection. It is well known that neutrophils are the main effector cells of the immune response contributing to bacterial clearance during a UTI^42^. However, migrated monocytes and differentiated macrophages have also been shown to influence the inflammatory response during a UTI^43,44^. NK-cells^45^, DCs^46^ and T-cells^47,48^ have all lately been implicated to be part of the host response during a UTI, but their specific contribution to the host response remains to be determined. With these results in mind, we proceeded with evaluating the ability of renal fibroblasts and renal epithelial cells to induce neutrophil migration in response to CFT073. We found that both fibroblasts and epithelial cells induced a significantly increased neutrophil migration in response to CFT073. However, no difference between the cell types were found. There may be additional neutrophil chemotactic factors released from renal epithelial cells in response to CFT073, e.g. CXCL2, CXCL3, CXCL5 and leukotriene B4^41^ that could explain the lack of difference in migration. Taken together, our findings show that renal fibroblasts are strong immunoreactive cells that have the same capacity as renal epithelial cells to recruit neutrophils in response to a UPEC infection.

This study provides novel insight into the role renal fibroblasts have during a UPEC infection. The ability to sense UPEC and mobilize a host response with neutrophil migration suggests that renal fibroblasts are not only structural cells that produce and regulate the extracellular matrix, but also highly immunoreactive cells. The ability of UPEC to invade renal fibroblasts may be one of the strategies utilized by the bacteria to subvert the host responses on its path to the bloodstream. Understanding how UPEC modulate our immune system in the kidneys may help us develop novel treatment strategies to prevent the bacteria from reaching the bloodstream.

## Methods

### Cell and bacterial culture

Primary human renal fibroblasts (Pelobiotech GmbH, Planegg, Germany) were derived from a healthy woman and the human renal epithelial cell line A498 (HTB-44, ATCC) was derived from a kidney carcinoma. Both cell lines were cultured in Dulbecco’s modified eagle medium (DMEM, Lonza, Basel, Switzerland) containing 10% foetal bovine serum (FBS), 2 mM L-glutamine, 1 mM non-essential amino acids, 50 U/mL penicillin and 50 ml/mL streptomycin (all from Invitrogen Ltd., Paisley, UK) at 37 °C in a 5% CO_2_ atmosphere. The cells were serum starved overnight in DMEM containing 2 mM L-glutamine, 1 mM non-essential amino acids and 50 µg/ml Gentamicin (Sigma-Aldrich, St. Louis, MO, USA). During experiments, the medium was replaced with DMEM containing 1% FBS, 1 mM non-essential amino acids and 2 mM L-glutamine. The UPEC strain CFT073 is isolated from a patient with pyelonephritis and urosepsis^49^. The UPEC strain UTI89 is isolated from a patient with an acute bladder infection^50^. MG1655 is a non-pathogenic *E*. *coli* K-12 strain^51^. The bacteria were maintained on tryptic soy agar and grown in Lysogeny broth (Difco Laboratories, Detroit, MI, USA) overnight on shake at 150 rpm 37 °C prior to experiments.

### Stimulation of renal fibroblasts and renal epithelial cells

The renal fibroblasts were stimulated with UTI89, MG1655, CFT073 wild-type and mutants at a multiplicity of infection (MOI) of 10, adenosine triphosphate (100 µM, ATP; Sigma-Aldrich), Adenosine (100 µM, Sigma-Aldrich), lipopolysaccharide (1 µg/ml, ultrapure LPS-B5, Invivogen, San Diego, CA, USA), flagellin (1 µg/ml, Invivogen) and Pam3CSK4 (1 µg/ml, Enzo Life Sciences, Lausen, Switzerland) for 6 hours and incubated at 37 °C with 5% CO_2_. The fibroblasts were also pre-incubated with DMSO (vehicle), p38 MAPK inhibitor SB203580 (10 µM, Santa Cruz Biotechnology Inc., Heidelberg, Germany), ERK1/2 inhibitor PD98059 (10 µM, Santa Cruz Biotechnology Inc), NF-κB inhibitor BAY 11-7082 (5 µM, Enzo Life Sciences) and the PKC inhibitor bisindolylmaleimide I (10 µM, Santa Cruz Biotechnology Inc) for 1 hours prior to CFT073 stimulation for 6 hours at MOI 10. Supernatants and mRNA were collected and kept at −80 °C until further analysis.

### RNA preparation and microarray

Total RNA was isolated from the primary renal fibroblasts using the RNeasy Mini Kit (Qiagen Technologies, Hilden, Germany) according to manufacturer instructions. RNA quality and integrity was analysed using Agilent 2100 Bioanalyzer (Agilent Technologies, Palo Alto, CA, USA) according to manufacturer instructions. The RNA integrity number (RIN) was above 9 for all samples. Total RNA was used to prepare labelled cRNA with the Low Input Quick Amp WT Labelling Kit (Agilent) according to manufacturer instructions. Hybridization of the labelled cRNA samples were done in a G2545A hybridization oven (Agilent) onto Agilent SurePrint G3 Human Gene Expression 8 × 60 k (Agilent Technologies) glass arrays according to manufacturer instructions and subsequently scanned with a G2505C array laser scanner (Agilent Technologies). Feature Extraction Software (version 10.7.3.1, Agilent Technologies) was used for image analysis and data extraction. Gene expression data is available in the GEO database with the accession number GSE124917.

### Quantitative Real-Time PCR (qPCR)

cDNA synthesis (100 ng of total RNA) was conducted using the High Capacity cDNA Reverse Transcription Kit (Applied Biosystems, CA, USA). Maxima SYBR Green qPCR Master Mix (ThermoFisher Scientific, MA, USA) was used for the real time-qPCR. 250 nM of primer (Supplementary Table S7, Eurofins MWG Synthesis GmbH, Ebersberg, Munich, Germany) and 5 ng of templet cDNA was used in the real time-qPCR. A CFX96 Touch™Real-Time PCR Detection System (Biorad, CA, USA) was used for the amplification using the following protocol: initial denaturation at 95 °C for 10 minutes, 40 cycles of denaturation at 95 °C for 15 seconds followed by annealing at 60 °C for 30 seconds and extension at 72 °C for 30 seconds. The qPCR was followed by a dissociation curve analysis between 60 and 95 °C. The Ct values were analysed by the ΔΔCt method and normalized to the endogenous control GAPDH (Glyceraldehyde 3-phosphate dehydrogenase). Fold difference was calculated as 2^−ΔΔCt^.

### Measurement of IL-8, CXCL10 and LDH release from primary human renal fibroblasts

Supernatants were collected after bacterial stimulation of renal fibroblasts and stored at −80 °C. An enzyme-linked immunosorbent assay (ELISA) was performed to measure IL-8 and CXCL10 release from the renal fibroblasts (ELISA MAX™ Deluxe Sets, BioLegend, San Diego, CA, USA) according to the manufacturer’s instructions and measured on a spectrophotometer (Multiskan Ascent, Thermo Labsystems, Helsinki, Finland). Cell viability was assessed by Pierce LDH cytotoxicity assay (Thermo Fisher Scientific) according to the manufacturer’s instructions. Cell viability was above 95% after UPEC infection.

### Adhesion and invasion assay

Renal fibroblasts were infected with wild-type CFT073, CFT073 deletion mutants CFT073ΔfimH and CFT073Δpap (eGFP) at MOI 10 and incubated at 37 °C with 5% CO_2_ for 3 hours to measure adhesion. The wells were then washed with PBS and the adhered eGFP expressing CFT073 were quantified and imaged with a Cytation 3 plate reader (BioTek, Winooski, VT, USA). Intracellular presence of bacteria was assessed by infecting bladder epithelial cells with UPEC at MOI 10 for 3 hours at 37 °C with 5% CO_2_. The wells were washed with PBS after infection and the medium was replaced with DMEM 1% FBS supplemented with 100 μg/ml gentamicin and incubated for 2 hours. The cells were thereafter washed again with PBS and lysed with 0.1% Triton-x 100 in PBS. The intracellular bacteria were plated on TSA plates, incubated at 37 °C overnight and CFU was counted. Intracellular presence of CFT073 was also assessed by infecting bladder epithelial cells with CFT073 at MOI 10 for 3 hours at 37 °C with 5% CO_2_. The cells were washed with PBS after infection and fixed for 15 minutes in 4% paraformaldehyde. 1% bovine serum albumin (BSA) was used to block unspecific binding. Extracellular CFT073 were labelled by incubation with a mouse monoclonal anti-*E*. *coli* LPS antibody (Abcam, Cambridge, UK) diluted 1:200 in PBS with 1% BSA for 1 hours at room temperature (RT). The cells were then washed and incubated with a secondary Rabbit anti-Mouse IgG Alexa Fluor® 594 (2 µg/ml, red fluorescence, Thermofisher, Massachusetts, USA) for 1 h at RT. Furthermore, intracellular CFT073 were labeled by permeabilization of fibroblasts with 0.1% Triton X-100 for 10 minutes, washed and reprobed with the mouse monoclonal anti-*E*. *coli* LPS antibody for 1 hour. CFT073 were stained with a secondary goat anti-mouse IgG-FITC conjugated antibody (Jackson ImmunoResearch Europe Ltd., Suffolk, UK) (green fluorescence) diluted 1:400 for 1 hour at RT. The nucleus was stained with 4′, 6-diamidino-2-phenylindole (DAPI, Bioledgend). CFT073 that stained both red and green were scored as extracellular adherent bacteria. Intracellular bacteria were only stained green. Images were obtained using an Olympus BX53 fluorescence microscope equipped with an Olympus DP74 camera.

### OLINK multiplex protein assay

Renal fibroblasts and renal epithelial cells (A498) were infected with CFT073 at MOI 10 and incubated at 37 °C with 5% CO_2_ for 6 hours. A panel of 92 inflammatory proteins were analysed simultaneously in the supernatants using the proximity extension assay on the Proseek Multiplex inflammation panel (Olink Bioscience, Uppsala, Sweden). The protein data is reported as normalized protein expression units where a high value corresponds to high protein concentration.

### Neutrophil isolation and migration assay

Human neutrophils were isolated from healthy blood donors by density gradient centrifugation of polymorphprep and lymphoprep reagents (AXIS-SHIELD PoC AS, Oslo, Norway) according to the manufacturer’s. An ethical approval has been granted by the regional ethics review board in Uppsala, Sweden (Dnr 2015/437), to isolate blood from healthy individuals after informed consent. Blood from healthy donors were collected according to the ethical guidelines of both the Declaration of Helsinki and the Swedish national board of health and welfare. The viability of the neutrophils was >90% as determined by the trypan blue exclusion test. Renal fibroblasts and renal epithelial cells (A498) were infected with CFT073 at MOI 10 and incubated at 37 °C with 5% CO_2_ for 6 h. Supernatants were collected and centrifuged for 5 minutes at 5000 × g to get rid of the bacteria. The bacteria free supernatants were added to the bottom well of a 3 μm pore size transwell system and 1·10^6^ neutrophils were added to the top well. Neutrophils were collected from the bottom well after 3 h of incubation and counted in a Bürker chamber.

### Statistical analysis and microarray data processing

The differences between groups assay were analysed with one-way ANOVA followed by Bonferroni test. Differences were considered statistically significant when p < 0.05. Data are presented as mean ± SEM, n = number of independent experiments. Microarray analysis was performed using Gene Spring GX version 12.0 (Agilent Technologies) after per chip and gene 75^th^ percentile shift normalization of samples. Different gene expression between groups was analysed with one-way ANOVA. Significantly expressed genes (p < 0.05) was obtained by Tukey HSD post-hoc test followed by Bonferroni multiple testing correction with a fold change set at ≥2. GO enrichment and KEGG pathway analysis were conducted with STRING (version 10.5) and the significance was set at a p-value < 0.05.

## Supplementary information

Supplementary information

Supplementary Table S1

Supplementary Table S2

Supplementary Table S3

Supplementary Table S4

Supplementary Table S5

Supplementary Table S6
