# Supplementary material for: Human renal fibroblasts are strong immunomobilizers during a urinary tract infection mediated by uropathogenic Escherichia coli
Source: Sci Rep. 2019 Feb 19;9:2296. doi: 10.1038/s41598-019-38691-8 (PMC6381130; doi:10.1038/s41598-019-38691-8)
Supplement: Supplementary file 1 — Supplementary information [file 41598_2019_38691_MOESM1_ESM.docx]

Supplementary information

**Human renal fibroblasts are strong immunomobilizers during a urinary tract infection mediated by uropathogenic *Escherichia coli***

Kristin Klarström Engström^1^, Boxi Zhang ^2^, Isak Demirel ^3,4*^

*^1^Department of Clinical trial unit, Faculty of Medicine and Health, Örebro University, Sweden;* *^2^Department of Physiology and Pharmacology, Karolinska Institutet, Stockholm, Sweden; ^3^iRiSC - Inflammatory Response and Infection Susceptibility Centre, Faculty of Medicine and Health, Örebro University, Sweden; ^4^ School of Medical Sciences, Örebro University, Örebro, Sweden*

***Corresponding author**: Isak Demirel PhD, School of Medical Sciences, Campus USÖ, Örebro University, SE-701 82 Örebro, Sweden. Phone: +4619303000. Email: [Isak.demirel@oru.se](mailto:Isak.demirel@oru.se)


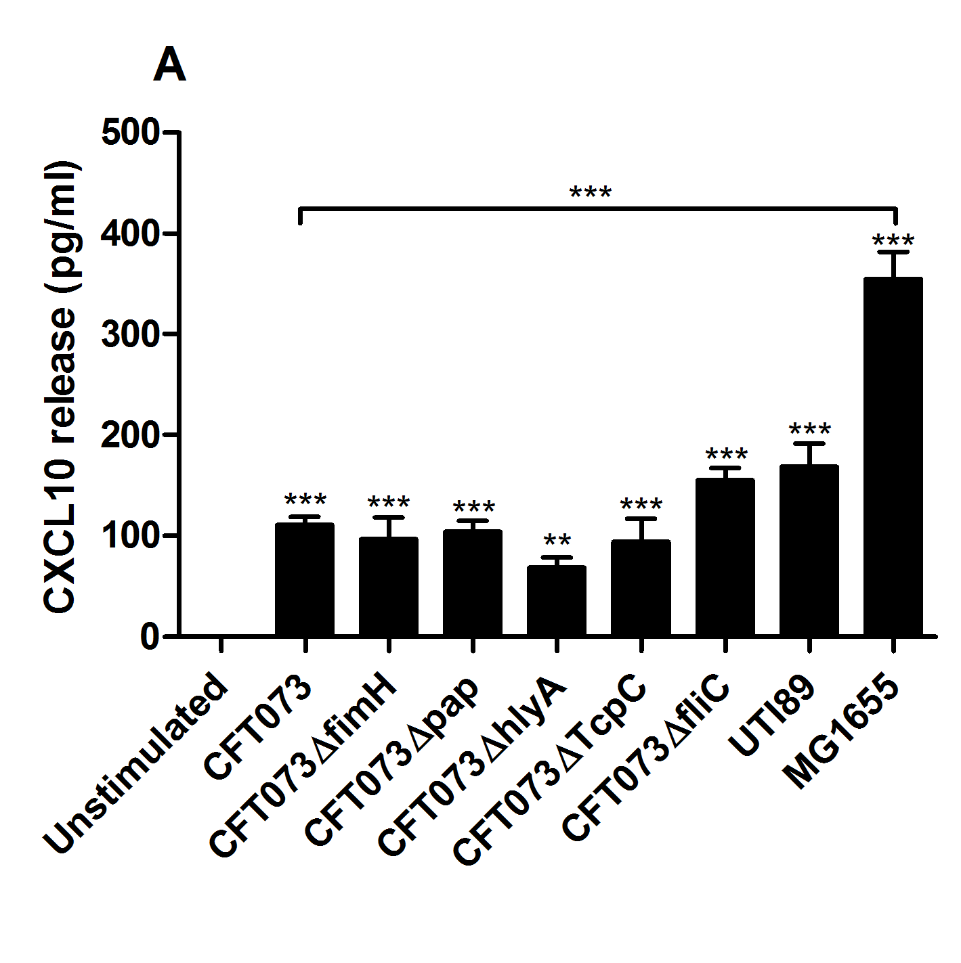


**Supplementary Figure S1. CXCL10 release from renal fibroblasts.** Primary human renal fibroblasts were stimulated with UTI89, MG1655, CFT073, CFT073Δpap, CFT073ΔfimH, CFT073ΔhlyA, CFT073ΔTcpC and CFT073ΔfliC at MOI 10 for 6 hours. Data are presented as mean ± SEM (n = 4 independent experiments). Asterisks denote statistical significance compared to respective unstimulated control cells (**p < 0.01, ***p < 0.001).

**Supplementary Table 7*.* Primers used for quantitative real-time PCR.**

| **Gene symbol** | **Oligonucleotide sequences (5´-3´)** |
| --- | --- |
| IL-1β | *F:* CCACAGACCTTCCAGGAGAATG  *R:* GTGCAGTTCAGTGATCGTACAGG |
| *NOD2* | *F:* GCACTGATGCTGGCAAAGAACG  *R:* CTTCAGTCCTTCTGCGAGAGAAC |
| *CXCL10* | *F:* GGTGAGAAGAGATGTCTGAATCC  *R:* R) GTCCATCCTTGGAAGCACTGCA |
| *CXCL9* | *F:* CTGTTCCTGCATCAGCACCAAC  *R:* TGAACTCCATTCTTCAGTGTAGCA |
| *PTCH1* | *F:* GCTGCACTACTTCAGAGACTGG  *R:* CACCAGGAGTTTGTAGGCAAGG |
| *TET1* | *F:* CAGGACCAAGTGTTGCTGCTGT  *R:* GACACCCATGAGAGCTTTTCCC |
| *PLCB2* | *F:* CCTGGAAGTGACGGCTTATGAG  *R:* GCTCTGTGAAGGACGAGATGAC |
| *CPEB1* | *F:* TGGCAGCCATCTTGAACGACCT  *R:* GCTGACTGCTTTCAGGTAACTCC |
| *GAPDH* | *F:* GTCTCCTCTGACTTCAACAGCG  *R:* ACCACCCTGTTGCTGTAGCCAA |
